# To assess the impact of individualized strategy and continuous glucose monitoring on glycemic control and mental health in pregnant women with diabetes

**DOI:** 10.3389/fendo.2025.1470473

**Published:** 2025-06-18

**Authors:** Mengxue Liu, Tong Chen, Shuai Wang, Na Li, Dan Liu

**Affiliations:** ^1^ Department of Endocrinology and Metabolism, First Affiliated Hospital of Dalian Medical University, Dalian, Liaoning, China; ^2^ Department of Health Services and Management, Dalian Neusoft University of Information, Dalian, Liaoning, China

**Keywords:** diabetes in pregnancy, gestational diabetes mellitus, continuous glucose monitoring, anxiety, depression, quality of life

## Abstract

**Objective:**

To assess the impact of individualized strategy and continuous glucose monitoring (CGM) on glycemic control and mental health(anxiety, depression, pregnancy-related anxiety and diabetes specific quality of life during pregnancy) in patients with diabetes in pregnancy (DIP).

**Methods:**

In this study, 80 pregnant women diagnosed with type 2 diabetes mellitus (T2DM) complicated with pregnancy or gestational diabetes mellitus (GDM) were enrolled. Participants were randomly assigned to either CGM group or self-monitoring of blood glucose (SMBG) group. Blood glucose was regularly monitored for 14 days to guide and adjust hypoglycemic treatment (lifestyle or hypoglycemic agents) of the patients in time. Baseline characteristics were collected after enrollment. Self-rating anxiety scale (SAS), self-rating depression scale (SDS), pregnancy-related anxiety questionnaire (PAQ), diabetes specific quality of life scale (DSQL) were used to evaluate the anxiety, depression, pregnancy-related anxiety and quality of life. Glycemic parameters and scale scores were collected before and after individualized strategy.

**Results:**

FBG and 2hPBG significantly decreased post-intervention in both groups (P<0.001). In the CGM group, the scores of SAS (39.59 ± 7.10 vs 37.15 ± 6.28), PAQ (24.15 ± 6.45 vs 22.59 ± 5.65) and DSQL (47.44 ± 9.01 vs 43.20 ± 9.00) after individualized strategy were significantly lower than those before individualized strategy (*P*<0.05). The SAS scale scores and PAQ scale scores were positively correlated with blood glucose levels (*P*<0.05).

**Conclusion:**

The individualized strategy encompasses an insulin titration protocol guided by CGM, coupled with structured lifestyle modifications that address dietary patterns, physical activity and more, combined with short-term glucose monitoring can exert a positive effect on glycemic improvement in the short term and meet the requirements of glycemic control in pregnancy, which has important clinical significance. The combined use of individualized strategy and CGM improves glycemic control and may have protective effects on psychological well-being.

**Clinical Trial Registration:**

https://www.chictr.org.cn, identifier ChiCTR2200060719.

## 1 Introduction

Diabetes in pregnancy (DIP) is a condition characterized by abnormal glucose metabolism during pregnancy, which includes both pregestational diabetes mellitus (PGDM) and gestational diabetes mellitus (GDM). PGDM denotes that a pregnant woman was diagnosed with diabetes mellitus (DM) prior to pregnancy. GDM is defined as the first occurrence or detection of impaired glucose tolerance during pregnancy. The prevalence of DIP in the U.S. ranges from 6.0% to 9.0%, with GDM constituting 90.0% of cases (1). According to the diagnostic criteria of the International Diabetes and pregnancy Research Group (IADPSG), a study in China in 2013 showed that the incidence of GDM was 17.5% (2). Hyperglycemia in pregnancy can lead to a variety of adverse pregnancy outcomes, such as macrosomia, shoulder dystocia, stillbirth, neonatal respiratory distress syndrome, neonatal hypoglycemia, etc. (1, 3), and is associated with an increased risk of maternal and fetal long-term complications such as type 2 diabetes mellitus (T2DM) (4, 5).

Pregnant women are more likely to be affected psychologically due to changes in physical and social psychological state, with anxiety and depression being more common (6). The global prevalence of prenatal anxiety and depression varied from 6.0% to 57.0% and 8.5% to 44.4%, respectively (7–9). On the other hand, anxiety and depression can cause hypothalamus-pituitary-adrenal dysfunction and then cause abnormal glucose tolerance or insulin resistance (IR) through sympathetic nerve activation (10, 11). GDM is more prone to anxiety and depression (12, 13). Hyperglycemia during pregnancy contributes to anxiety and depression through multiple mechanisms, including lack of awareness of the disease, worry about the health problems of future generations, stress response and so on. Anxiety can affect the mother’s emotional balance and fetal development, and it can also lead to low birth weight, premature birth and other adverse pregnancy outcomes (14–16). Therefore, it is crucial to pay attention to the psychological status of patients with DIP. It is worth mentioning that PGDM and GDM are significantly different in terms of clinical manifestations, treatment measures and clinical prognosis. In addition, the characteristics of glucose metabolism are different at different gestational ages, so it is necessary to analyze the difference of mental health status between them.

Blood glucose monitoring plays an indispensable role in the blood glucose strategy of patients with DM. The most widely used blood glucose monitoring method in a clinic is self-monitoring of blood glucose (SMBG), based on capillary glucose testing. Whereas SMBG can only reflect the instantaneous capillary blood glucose level at that time but cannot recall the overall trend and fluctuation of blood glucose, and some patients cannot stand the pain of fingertip blood glucose monitoring and the economic burden related to blood glucose monitoring. Continuous glucose monitoring (CGM) is a new blood glucose monitoring method that has been used in clinics in recent years, which reflects the whole-day blood glucose level and blood glucose fluctuation by measuring the blood glucose concentration in tissue fluid. The daily glucose trend chart, glucose fluctuation trend and other related data can be obtained to encourage both doctors and patients to evaluate the blood glucose more thoroughly and assist in adjusting of the hypoglycemic treatment to achieve the targets of blood glucose control. CGM and SMBG were used in this study to better understand the blood glucose level of patients with DIP, provide reference for individualized strategy and evaluate the efficacy, anxiety, depression and quality of life of individualized strategy combined with blood glucose monitoring.

## 2 Materials and methods

### 2.1 Participants and study design

**Figure 1:**
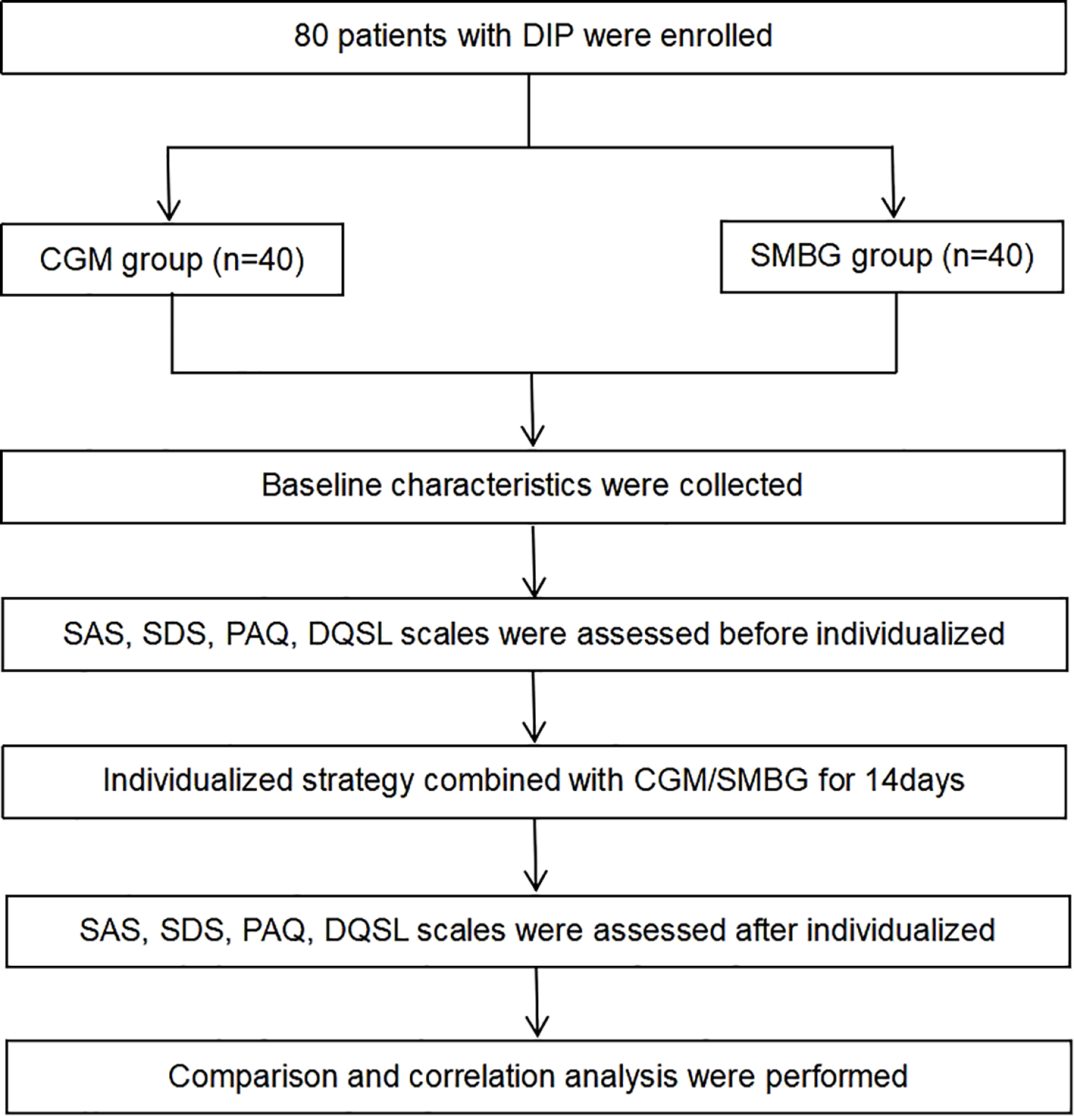
Flowchart of material and methods.

This study enrolled 80 pregnant women who met the diagnosis of DIP (including PGDM and GDM) in the outpatient clinic of the First Affiliated Hospital of Dalian Medical University from June 2022 to July 2022 (**Figure 1**). This study was approved by the ethics committee of the First Affiliated Hospital of Dalian Medical University. The inclusion criteria included: 1) 18–45 years old; 2) singleton pregnancy; 3) no previous history of mental illness; 4) voluntary use of CGM or SMBG, who have good understanding and communication skills. The exclusion criteria included: 1) anxiety or depression diagnosed before pregnancy; 2) recently experienced severe stress events, complicated with infection, heart failure, kidney insufficiency, or other serious complications; 3) poor compliance. The participants were randomly divided into the CGM group and the SMBG group. The pregnant women in the CGM group used FreeStyle Libre (Abbott Diabetes Care Ltd) to dynamically monitor their blood glucose for 14 consecutive days. By using the scanner, patients can obtain an immediate glucose value, nearly 8 hours of glucose data and a glucose change trend, and the system can automatically save an average of blood glucose every 15 minutes, recording a total of 96 blood glucose values per day, and finally obtaining a 14-day glucose trend chart. The pregnant women in the SMBG group, on the other hand, utilized a home blood glucose meter to track changes in peripheral blood glucose, and recorded fasting and 2-hour postprandial blood glucose for 14 days.

Fasting blood glucose (FBG) and 2 hours postprandial blood glucose (2hPBG) were recorded using CGM or SMBG. To minimize bias, all participants received standardized instructions from the same endocrinologist. Patients sent recorded blood glucose and daily exercise and diet information to the endocrinologist every 1 to 3 days through the WeChat app (application) during the study period. The endocrinologist then gives patients timely lifestyle advice, including diet, exercise instructions and insulin dose adjustments, based on blood glucose control targets during pregnancy. According to American Diabetes Association, blood glucose targets during pregnancy: FPG or pre-prandial blood glucose ≤ 5.3mmol/L, 1h post-prandial ≤ 7.8mmol/L, 2h post-prandial ≤ 6.7mmol/L.

### 2.2 Hypoglycemic treatment guidance

The dietary principle is the principle of low-glycemic load. Maintain weight gain within a reasonable range through a low-glycemic load diet to avoid hypoglycemia, hyperglycemia and diabetic ketosis. Individual nutrient intake includes: 1) protein: ensure adequate intake of high-quality protein, such as eggs, skim milk, fish and shrimp, beef, mutton, pork, tofu, skinless poultry, etc., which are conducive to the growth and development of the fetus. 2) fat: a high-fat diet is challenging to digest and increases the burden of insulin; limit the intake of high-fat and high-cholesterol foods. 3) carbohydrates: regular and quantitative, preferably buckwheat, oats, whole wheat, brown rice and other hypoglycemic effects; eat less stuffing, noodles, porridge, etc.; avoid desserts, sweets, drinks and excessive intake of fruits rich in monosaccharides. 4) inorganic salts and vitamins: vegetables, nuts, fruits and lean meat are recommended as sources of vitamins, calcium, magnesium and trace elements.

Reasonable diet combined with personalized exercise can effectively reduce blood glucose. Before instructing pregnant women to exercise, first exclude patients with contraindications for exercise during pregnancy, such as heart disease, threatened premature delivery, low progesterone, threatened abortion, fetal intrauterine growth restriction, placental abnormalities, cervical dysfunction, etc. The recommended way of exercise is aerobic exercise such as walking, exercise time in half an hour to one hour after meal, the duration of activity is about 20–30 minutes, to avoid hypoglycemia caused by excessive activity.

After lifestyle interventions, patients with DIP who still failed to achieve blood glucose control targets were treated with insulin based on lifestyle modification. The first choice for basic insulin is hypodermic injection of insulin detemir before bedtime. Insulin glargine or intermediate-acting insulin (Novolin N) should be used instead in the event of an allergic reaction. In addition, the preferred pre-prandial short-acting insulin is insulin aspart subcutaneously injected 5 minutes before meals, and insulin lispro should be used if allergic. During pregnancy, the insulin dose was modified based on blood glucose control targets.

### 2.3 Baseline characteristics

Baseline characteristics of DIP patients were collected, including age, gestational age, prepregnancy body mass index (BMI), pregnancy type (primipara or multipara), history of abortion, family history of diabetes, glycosylated hemoglobin (HbA1c), hypoglycemic regimen.

### 2.4 Assessment of glycemic control

FBG and 2hPBG were collected from patients with DIP before and after individualized strategy. CGM-measurements and glycemic variability parameters included time in range (TIR), time above range (TAR), time below range (TBR), average glucose (AG), estimated HbA1c, standard deviation of blood glucose (SDBG), mean amplitude of glucose excursions (MAGE) and coefficient of variation (CV) were also collected.

### 2.5 Assessment of anxiety, depression and quality of life

Anxiety, depression, pregnancy-related anxiety and diabetes specific quality of life scales were assessed in patients before and after individualized strategy respectively. In this study, patients’ anxiety, depression and quality of life were evaluated by applying self-rating anxiety scale (SAS) (17), self-rating depression scale (SDS) (18), pregnancy-related anxiety questionnaire (PAQ) (19), diabetes specific quality of life scale (DSQL) (20). These scales have been transformed into Chinese versions and are widely used in China with good reliability and validity (21–23). There are 20 items in SAS and SDS, respectively. SAS standard points ≥ 50 were anxiety and SDS standard points ≥ 53 were depression, according to Chinese norms. The PAQ scale, compiled by Chinese scholars, has a total of 13 items, including three aspects of pregnant women worried about fetal health, delivery process and self-care. The total score ≥ 24 was pregnancy-related anxiety, with a higher total score indicating a higher level of pregnancy-related anxiety. The DSQL scale evaluated the quality of life of patients with DIP from physical, psychological, social relations and treatment dimensions, a total of 27 items, with a lower total score indicating a higher level of quality of life.

### 2.6 Statistical analysis

Statistical analysis was performed using Statistical Package of Social Sciences (SPSS) 26. Data normality was evaluated before using parametric tests. Data with normal distribution were expressed by mean ± standard deviation ( $\bar{x}$ ± s), and data with non-normal distribution were expressed by medians, and count data were expressed by [n (%)]. T-test was used for continuous variables to compare the difference between the two groups, chi-square test and Fisher’s exact test were used for categorical variables to compare the difference between the two groups. Within-group differences were compared with paired t-test. Pearson correlation analysis was used to analyze the correlation between scale score and other data. All the tests were performed by two-sided test, with a *P* value<0.05 as the statistical difference evaluation standard.

## 3 Results

A total of 80 eligible women completed study, including 40 women in the CGM group and 40 women in the SMBG group. Among the 80 participants, 32 had PGDM (all with T2DM), while 48 had GDM.

### 3.1 Baseline characteristics

#### 3.1.1 Comparison of baseline characteristics between the CGM group and the SMBG group

The mean age of DIP patients was 32.90 ± 4.13 years old, and there were 27 (33.75%) patients ≥ 35 years old. The mean pre-pregnancy BMI of the patients was 27.22 ± 4.80kg/m^2^, and there were 49 patients (61.3%) with pre-pregnancy BMI>25kg/m^2^. There were 51 patients (63.8%) with a family history of diabetes. There were no statistically significant differences between the CGM group and the SMBG group in age, gestational age, pre-pregnancy BMI, type of DIP, pregnancy type, proportion of abortion history, proportion of family history of diabetes, FBG, 2hPBG, hypoglycemic treatment and HbA1c (*P*>0.05) (**Table 1**). There was no difference in baseline between the two groups, indicating comparability of data between the two groups.

**Table 1:** Comparison of baseline characteristics between the CGM group and the SMBG group.

Parameters	CGM group (n=40)	SMBG group (n=40)	*P* value
Age (years)	33.38 ± 3.89	32.43 ± 4.36	0.307
Gestation age (weeks)	22.25 ± 8.47	20.95 ± 8.31	0.490
Prepregnancy BMI (kg/m^2^)	27.12 ± 4.51	27.32 ± 5.13	0.854
Type [n (%)]			0.648
PGDM	17 (42.50%)	15 (34.50%)	
GDM	23 (57.50%)	25 (62.50%)	
Pregnancy type [n (%)]			0.152
Primipara	24 (60.0%)	30 (75.0%)	
Multipara	16 (40.0%)	10 (25.0%)	
History of abortion [n (%)]	20 (50.0%)	18 (45.0%)	0.654
Family history of diabetes [n (%)]	25 (62.5%)	26 (65.0%)	0.816
FBG (mmol/L)	6.81 ± 2.38	6.74 ± 1.49	0.867
2hPBG (mmol/L)	9.89 ± 3.11	8.86 ± 2.76	0.121
HbA1c (%)	6.55 ± 1.80	6.27 ± 1.26	0.431
Hypoglycemic treatment			0.491
Insulin	26 (65.0%)	23 (57.5%)	
Lifestyle	14 (35.0%)	17 (42.5%)	

Data are presented as mean ± SD or number (%).

CGM, continuous glucose monitoring; SMBG, self-monitoring of blood glucose; BMI, body mass index; PGDM, pregestational diabetes mellitus; GDM, gestational diabetes mellitus; FBG, fasting blood glucose; 2hPBG, 2 hours postprandial blood glucose; HbA1c, glycosylated hemoglobin.

#### 3.1.2 Comparison of baseline characteristics between PGDM patients and GDM patients

There were 32 patients (40.0%) with PGDM and 48 patients (60.0%) with GDM. There were no significant differences in age, pre-pregnancy BMI, pregnancy type and proportion of abortion history between PGDM patients and GDM patients (*P*>0.05). The gestational age of PGDM patients was smaller than that of GDM patients, while the proportion of family history of diabetes, FBG, 2hPBG, HbA1c and the proportion of insulin used were significantly higher than those of GDM patients, with statistical significance (*P*<0.05) (**Table 2**). This indicated that PGDM presents more significant blood glucose fluctuations and more severe hyperglycemia compared to GDM.

**Table 2:** Comparison of baseline characteristics between PGDM and GDM patients.

Parameters	PGDM (n=32)	GDM (n=48)	*P* value
Age (years)	32.28 ± 3.95	33.31 ± 4.24	0.227
Gestation age (weeks)	18.50 ± 7.73	23.67 ± 8.20*	0.006
Prepregnancy BMI (kg/m^2^)	28.11 ± 4.55	26.62 ± 4.92	0.175
Pregnancy type [n (%)]			0.436
Primipara	20 (62.50%)	34 (70.83%)	
Multipara	12 (37.50%)	14 (29.17%)	
History of abortion [n (%)]	16 (50%)	22 (45.83%)	0.715
Family history of diabetes [n (%)]	27 (84.375%)	24 (50%)*	0.002
FBG (mmol/L)	7.92 ± 2.31	6.01 ± 1.23*	<0.001
2hPBG (mmol/L)	10.82 ± 3.35	8.42 ± 2.26*	<0.001
HbA1c (%)	7.40 ± 1.89	5.75 ± 0.76*	<0.001
Hypoglycemic treatment			<0.001
Insulin	31 (96.875%)	18 (37.5%)	
Lifestyle	1 (3.125%)	30 (62.5%)	

Data are presented as mean ± SD or number (%).

PGDM, pregestational diabetes mellitus; GDM, gestational diabetes mellitus; BMI, body mass index; FBG, fasting blood glucose; 2hPBG, 2 hours postprandial blood glucose; HbA1c, glycosylated hemoglobin. *P value<0.05 was considered significant.

### 3.2 Glycemic parameters

#### 3.2.1 Comparison of glycemic parameters before and after individualized strategy

FBG and 2hPBG of the two groups after individualized strategy by different blood glucose monitoring methods (CGM or SMBG) were significantly lower than those before individualized strategy (*P*<0.001) (**Table 3**), indicating that individualized strategy exerted a positive effect on glycemic improvement in the short term.

**Table 3:** Comparison of FBG and 2hPBG before and after individualized strategy.

Blood Glucose	CGM group (n=40)			SMBG group (n=40)		
	before	after	*P* value	before	after	*P* value
FBG (mmol/L)	6.81 ± 2.38	5.39 ± 0.63*	<0.001	6.74 ± 1.49	5.71 ± 1.04*	<0.001
2hPBG (mmol/L)	9.89 ± 3.11	6.87 ± 0.96*	<0.001	8.86 ± 2.76	6.95 ± 1.23*	<0.001

Data are presented as mean ± SD.

CGM, continuous glucose monitoring; SMBG, self-monitoring of blood glucose; FBG, fasting blood glucose; 2hPBG, 2 hours postprandial blood glucose; *P value<0.05 was considered significant.

#### 3.2.2 Glycemic variability parameters

Compared with GDM patients, glycemic variability parameters calculated by CGM included TAR, AG, estimated HbA1c, SDBG and MAGE of PGDM patients were significantly higher and TBR was significantly lower (*P*<0.05); there were no significant difference in TIR and CV between PGDM and GDM (*P*>0.05) (**Table 4**). PGDM exhibited higher blood glucose than GDM, with significant blood glucose fluctuation and more severe hyperglycemia.

**Table 4:** Comparison of glycemic variability parameters between PGDM patients and GDM patients in the CGM group.

Glycemic variability parameters	PGDM (n=17)	GDM (n=23)	*P* value
TIR (%)	87.00 (69.00, 92.00)	89.00 (71.00, 93.00)	0.741
TAR (%)	7.00 (4.00, 29.00)	1.00 (0, 9.00)*	0.013
TBR (%)	1.00 (0, 4.00)	9.00 (3.00, 27.00)*	0.006
AG (mmol/L)	6.71 ± 1.43	5.37 ± 0.76*	0.001
estimated HbA1c (%)	5.85 ± 0.92	5.01 ± 0.49*	0.002
SDBG (mmol/L)	1.61 ± 0.20	1.21 ± 0.29*	0.003
MAGE (mmol/L)	3.02 ± 0.15	2.44 ± 0.67*	0.003
CV (%)	24.90 ± 3.43	22.78 ± 3.14	0.158

Data are presented as mean ± SD, mean (interquartile range).

PGDM, pregestational diabetes mellitus; GDM, gestational diabetes mellitus; TIR, time in range; TAR, time above range; TBR, time below range; AG, average glucose; HbA1c, glycosylated hemoglobin; SDBG, standard deviation of blood glucose; MAGE, mean amplitude of glucose excursions; CV, coefficient of variation. *P value<0.05 was considered significant.

### 3.3 The score of SAS, SDS, PAQ and DSQL scales

Among patients with DIP, 7.5% had anxiety, 17.5% had depression, 5.0% had anxiety and depression, and 45.0% had pregnancy-related anxiety.

#### 3.3.1 Comparison of scale scores between the CGM group and the SMBG group before and after individualized strategy

Before and after individualized strategy, there were no significant differences in SAS, SDS, PAQ and DSQL scores between the CGM group and the SMBG group (*P*>0.05) (**Table 5**).

**Table 5:** Comparison of inter-group scale scores between the CGM group and the SMBG group before and after individualized strategy.

Scale	Before			After		
	CGM group (n=40)	SMBG group (n=40)	*P* value	CGM group (n=40)	SMBG group (n=40)	*P* value
SAS	39.75 ± 7.11	39.28 ± 8.54	0.788	37.35 ± 6.22	39.03 ± 7.90	0.296
SDS	43.45 ± 11.21	44.55 ± 8.92	0.629	42.20 ± 9.82	45.20 ± 8.66	0.151
PAQ	24.28 ± 6.48	24.18 ± 5.75	0.942	22.73 ± 5.65	22.30 ± 5.74	0.739
DSQL	47.73 ± 8.93	45.45 ± 7.57	0.223	43.40 ± 9.02	44.83 ± 9.43	0.492

Data are presented as mean ± SD.

CGM, continuous glucose monitoring; SMBG, self-monitoring of blood glucose; SAS, self-rating anxiety scale; SDS, self-rating depression scale; PAQ, pregnancy-related anxiety questionnaire; DSQL, diabetes specific quality of life scale..

**Figure 2:**
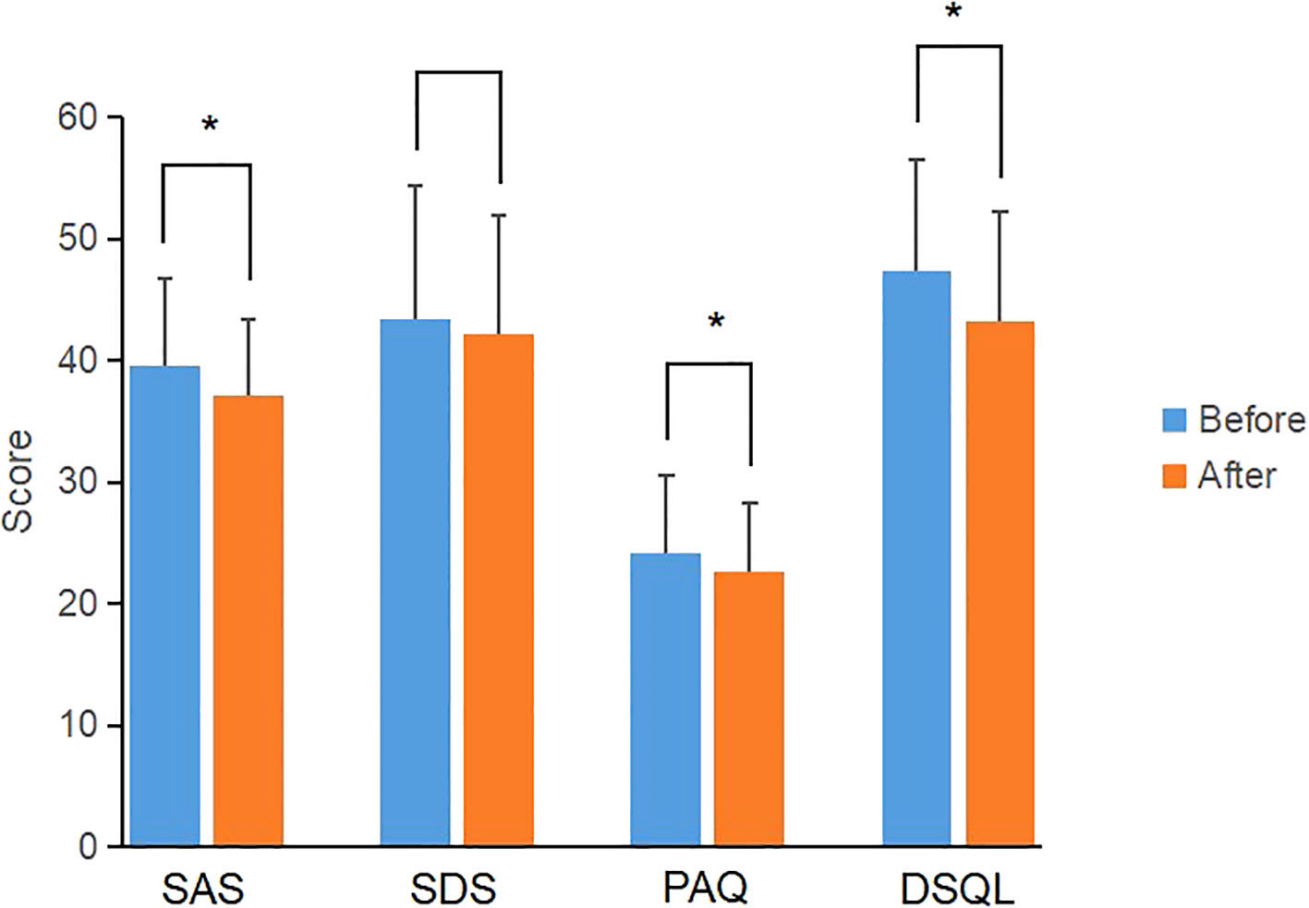
Comparison of scale scores of the CGM group before and after individualized strategy. *P value<0.05 was considered significant.

In the CGM group, the scores of SAS, PAQ and DSQL after individualized strategy were significantly lower than those before individualized strategy (*P*<0.05) (**Figure 2**); the SDS score were lower than that before individualized strategy, but the difference was not statistically significant (*P*>0.05) (**Table 6**). In the SMBG group, the scores of PAQ after individualized strategy were significantly lower than that before individualized strategy (*P*<0.05); the scores of SAS, SDS and DSQL scales after individualized strategy had no statistical difference compared with those before individualized strategy (*P*>0.05) (**Table 6**), indicating that CGM is superior to SMBG in improving anxiety and quality of life.

**Table 6:** Comparison of within-group scale scores between the CGM group and the SMBG group before and after individualized strategy.

Scale	CGM group (n=40)			SMBG group (n=40)		
	Before	After	*P* value	Before	After	*P* value
SAS	39.75 ± 7.11	37.35 ± 6.22*	0.003	39.28 ± 8.54	39.03 ± 7.90	0.802
SDS	43.45 ± 11.21	42.20 ± 9.82	0.157	44.55 ± 8.92	45.20 ± 8.66	0.558
PAQ	24.28 ± 6.48	22.73 ± 5.65*	0.020	24.18 ± 5.75	22.30 ± 5.74*	0.022
DSQL	47.73 ± 8.93	43.40 ± 9.02*	<0.001	45.45 ± 7.57	44.83 ± 9.43	0.575

Data are presented as mean ± SD.

CGM, continuous glucose monitoring; SMBG, self-monitoring of blood glucose; SAS, self-rating anxiety scale; SDS, self-rating depression scale; PAQ, pregnancy-related anxiety questionnaire; DSQL, diabetes specific quality of life scale. *P value<0.05 was considered significant.

#### 3.3.2 Comparison of scale scores between PGDM patients and GDM patients before and after individualized strategy

In the CGM or SMBG group, there were no statistical differences in SAS, SDS, PAQ and DSQL scale scores between PGDM patients and GDM patients in before and after individualized strategy (*P*>0.05) (**Table 7**).

**Table 7:** Comparison of inter-group scale scores between PGDM patients and GDM patients before and after individualized strategy.

Scale	CGM group (n=40)						SMBG group (n=40)					
	Before			After			Before			After		
	PGDM (n=17)	GDM (n=23)	*P* value	PGDM (n=17)	GDM (n=23)	*P* value	PGDM (n=15)	GDM (n=25)	*P* value	PGDM (n=15)	GDM (n=25)	*P* value
SAS	39.82 ± 7.11	39.70 ± 7.26	0.956	38.12 ± 6.85	36.78 ± 5.82	0.510	41.87 ± 8.04	37.72 ± 8.61	0.139	40.27 ± 7.05	38.28 ± 8.42	0.449
SDS	43.53 ± 12.48	43.39 ± 10.46	0.970	43.88 ± 11.16	40.96 ± 8.76	0.359	45.87 ± 9.52	43.76 ± 8.64	0.477	44.00 ± 8.86	45.92 ± 8.64	0.504
PAQ	24.41 ± 7.05	24.17 ± 6.18	0.910	22.82 ± 5.50	22.65 ± 5.87	0.926	24.73 ± 6.45	23.84 ± 5.40	0.640	21.73 ± 5.80	22.64 ± 5.80	0.635
DSQL	47.35 ± 8.98	48.00 ± 9.09	0.824	44.29 ± 11.41	42.74 ± 6.96	0.596	47.33 ± 7.04	44.32 ± 7.79	0.228	46.47 ± 10.47	43.84 ± 8.82	0.401

Data are presented as mean ± SD.

CGM, continuous glucose monitoring; SMBG, self-monitoring of blood glucose; PGDM, pregestational diabetes mellitus; GDM, gestational diabetes mellitus; SAS, self-rating anxiety scale; SDS, self-rating depression scale; PAQ, pregnancy-related anxiety questionnaire; DSQL, diabetes specific quality of life scale.

**Figure 3:**
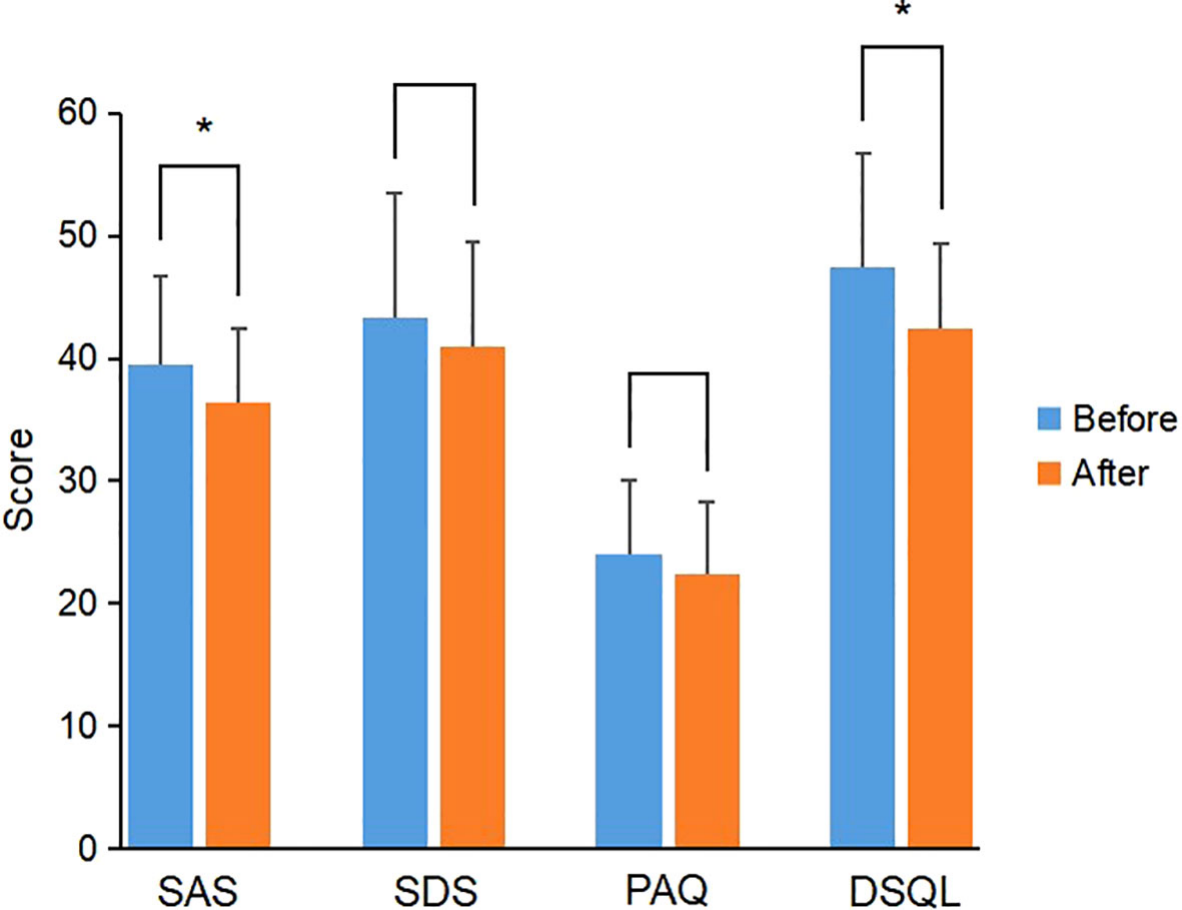
Comparison of scale scores of GDM patients before and after individualized strategy in the CGM group. *P value<0.05 was considered significant..

In the CGM group, the scores of SAS, SDS, PAQ and DSQL in PGDM patients after individualized strategy were not significantly different from those before individualized strategy, while the scores of SAS and DSQL in GDM patients were significantly lower than those before individualized strategy (*P*<0.05) (**Figure 3**); the scores of SDS and PAQ were lower than those before individualized strategy, but there was no significant difference (*P*>0.05) (**Table 8**).

**Table 8:** Comparison of within-group scale scores between PGDM patients and GDM patients before and after individualized strategy.

Scale	CGM group (n=40)						SMBG group (n=40)					
	PGDM (n=17)			GDM (n=23)			PGDM (n=15)			GDM (n=25)		
	Before	After	*P*	Before	After	*P*	Before	After	*P*	Before	After	*P*
SAS	39.82 ± 7.11	38.12 ± 6.85	0.176	39.70 ± 7.26	36.78 ± 5.82*	0.008	41.87 ± 8.04	40.27 ± 7.05	0.251	37.72 ± 8.61	38.28 ± 8.42	0.685
SDS	43.53 ± 12.48	43.88 ± 11.16	0.747	43.39 ± 10.46	40.96 ± 8.76	0.063	45.87 ± 9.52	44.00 ± 8.86	0.243	43.76 ± 8.64	45.92 ± 8.64	0.147
PAQ	24.41 ± 7.05	22.82 ± 5.50	0.158	24.17 ± 6.18	22.65 ± 5.87	0.069	24.73 ± 6.45	21.73 ± 5.80*	0.028	23.84 ± 5.40	22.64 ± 5.80	0.251
DSQL	47.35 ± 8.98	44.29 ± 11.41	0.144	48.00 ± 9.09	42.74 ± 6.96*	<0.001	47.33 ± 7.04	46.47 ± 10.47	0.715	44.32 ± 7.79	43.84 ± 8.82	0.674

Data are presented as mean ± SD.

CGM, continuous glucose monitoring; SMBG, self-monitoring of blood glucose; PGDM, pregestational diabetes mellitus; GDM, gestational diabetes mellitus; SAS, self-rating anxiety scale; SDS, self-rating depression scale; PAQ, pregnancy-related anxiety questionnaire; DSQL, diabetes specific quality of life scale. * *P* value<0.05 was considered significant.

In the SMBG group, the scores of PAQ in patients with PGDM were significantly lower than those before individualized strategy (*P*<0.05), while the scores of SAS, SDS, PAQ and DSQL in GDM patients after individualized strategy were not significantly different from those before individualized strategy (*P*>0.05) (**Table 8**). In addition, the degree of blood glucose elevation and fluctuation in patients with GDM is less than that in PGDM.

#### 3.3.3 Correlation analysis between SAS, SDS, PAQ, DSQL scale scores and other parameters before individualized strategy

The score of SAS scale was positively correlated with HbA1c, FBG in patients with DIP, and the score of PAQ scale was positively correlated with FBG, indicating that patients with higher blood glucose level tend to have higher anxiety scores. The scores of SDS and DSQL were not significantly correlated with gestational age, age, HbA1c, FBG, 2hPBG and prepregnancy BMI (**Table 9**).

**Table 9:** Correlation analysis between scale scores and other parameters.

Parameters	SAS		SDS		PAQ		DSQL	
	r	*P* value	r	*P* value	r	*P* value	r	*P* value
Gestation age	-0.212	0.059	0.006	0.961	-0.173	0.125	-0.106	0.350
Age	0.057	0.614	0.048	0.674	-0.083	0.464	-0.010	0.932
HbA1c	0.239	0.033	0.093	0.413	0.187	0.097	0.178	0.115
FBG	0.246	0.028	0.151	0.182	0.231	0.039	0.172	0.127
2hPBG	0.146	0.197	0.074	0.512	0.203	0.070	0.180	0.110
Prepregnancy BMI	-0.082	0.468	0.035	0.757	0.094	0.406	-0.020	0.861

SAS, self-rating anxiety scale; SDS, self-rating depression scale; PAQ, pregnancy-related anxiety questionnaire; DSQL, diabetes specific quality of life scale. HbA1c, glycosylated hemoglobin; FBG, fasting blood glucose; 2hPBG, 2 hours postprandial blood glucose; BMI, body mass index. *P* value<0.05 was considered significant.

## 4 Discussion

Due to physiological factors such as hormone fluctuations, as well as increased sensitivity to family, social and other factors, pregnant women are prone to adverse emotions such as anxiety and depression. In this study, the probabilities of anxiety, depression, anxiety and depression in DIP patients were 7.5%, 17.5% and 5.0%, respectively, which was similar to the probabilities of anxiety, depression, anxiety and depression in early pregnancy found by Tang et al. (7.7%, 10.5%, 4.8%) (24). But Other studies have shown that about 12.0% of pregnant women experience depression and up to 22.0% experience anxiety in late pregnancy (25, 26). Studies reported that the prevalence of maternal depression and anxiety was as high as 27.0% and 24.0%, respectively (27, 28). We emphasize the importance of routine psychological assessment and intervention in the management of DIP.

In addition, Compared with normal GDM pregnant women, GDM pregnant women with anxiety and depression are more prone to adverse outcomes in terms of blood glucose, delivery mode and maternal and infant outcomes during pregnancy (13). A study showed a significant increase in anxiety and depression symptoms among pregnant women during the COVID-19 pandemic, which could have long-term effects on their offspring (59). According to statistics, the probability of depression in patients with DM was 3 times higher than that in healthy people, and the incidence of depression in patients with T1DM was as high as 12.0% (29). Patients with T2DM had a high incidence of anxiety and depression, and patients with adverse emotions had poor compliance, which was detrimental to disease management (60).

Studies have shown that anxiety and depression may be risk factors for GDM (30, 31), but there is no unified conclusion on the correlation between anxiety, depression and GDM at present. Anxiety and depression can lead to hormone imbalance in the body, which seriously affects pregnancy outcomes and blood glucose control of GDM. In addition to physiological factors, psychological factors such as anxiety and depression are also important causes of GDM (32, 33). Anxiety and depression can lead to chronic hypothalamic-pituitary-adrenal (HPA) axis hyperfunction, resulting in increased cortisol release and IR (34), increasing the risk of GDM in pregnant women. At the same time, GDM increases the susceptibility of pregnant women to anxiety and depression, and the likelihood of prenatal or postpartum depression is 2–4 times higher than that without GDM (35–38), which may be related to their awareness of poor blood glucose control and pregnancy complications and adverse pregnancy outcomes (39, 40). However, some studies suggest that anxiety and depression do not increase the probability of GDM in pregnant women (41–44), nor does GDM increase the risk of prenatal or postpartum depression (45, 46). With the implementation of the three-child policy, it is urgent to pay attention to the psychological status of pregnancy and avoid adverse pregnancy outcomes under the guidance of demand. In addition, pregnancy-related anxiety refers to a kind of anxiety and painful emotional experience caused by pregnancy to pregnant women (47). Feng et al. found that pregnancy-related anxiety accounted for 59.1% of GDM patients (48). In this study, the incidence of pregnancy-related anxiety in patients with DIP was 45%, which was higher than that of 31% of normal pregnant women at mid-pregnancy and 29% at late-pregnancy (49).

One study identified three sources of anxiety and depression in patients with GDM: the diagnosis of GDM and perceptions of high-risk pregnancies; glycemic control during dietary intervention; the fear of maternal and infant complications. The study identified the fear of pregnancy complications as the most significant source of stress for GDM. In addition, pregnant women who received insulin treatment were more stressed than those who received dietary intervention only (45). This is consistent with the recent study of Lee et al. (50), which exacerbates patients’ concerns about treatment because of the relationship between insulin and hypoglycemia events. Horsch et al. believed that anxiety was related to FBG (3), which was consistent with the findings of this study that anxiety was positively correlated with HbA1c, FBG, and pregnancy-related anxiety was positively correlated with FBG and 2hPBG. Through the analysis of the three aspects of worrying about fetal health, delivery process and self-care contained in the PAQ scale, it was found that the pregnancy-related anxiety of DIP patients mainly originated from worrying about the physical health of the fetus.

Clinical application of CGM can reduce the risk of hypoglycemia and hyperglycemia as well as blood glucose variability and improve the quality of life of patients (51). CGM contributed to a significant improvement in diabetes specific quality of life in T1DM adults (52). However, one study suggested that the use of well-standardized, structured SMBG could reduce depressive symptoms in a large number of moderately depressed or distressed T2DM patients with poor glycemic control (53). There is no study to observe the effects of individualized strategy through CGM on anxiety, depression and quality of life in DIP patients. This study found that scores on the SAS and PAQ scales were positively correlated with blood glucose parameters, suggesting that effective glycemic control may play a crucial role in mitigating psychological distress in DIP patients. Additionally, this study verified that an individualized strategy combined with CGM can improve anxiety, pregnancy-related anxiety, and diabetes-specific quality of life. The reasons considered are mainly that CGM is easy to monitor blood glucose in patients, which can quickly and painlessly obtain blood glucose value, predict the trend of glucose change, timely detect occult blood glucose abnormalities (hyperglycemia or hypoglycemia), adjust lifestyle and hypoglycemic treatment and optimize treatment effects. Therefore, CGM shows advantages over traditional SMBG in improving anxiety and quality of life. However, no significant improvement in depression was found for the following reasons: the results of this study did not find any correlation between SDS scale scores and blood glucose parameters; there were many factors considering the causes of depression in DIP; the duration of monitoring blood glucose by CGM was short (14 days), and the effect of improving patients’ depression was limited in a short time. In addition, we also found that individualized strategy with CGM played a more significant role in improving anxiety and quality of life in patients with GDM compared with patients with PGDM, probably because most patients with PGDM had taken lifestyle intervention combined with oral drugs or insulin before pregnancy and had a certain degree of understanding of the disease. In addition, the degree of blood glucose elevation and blood glucose fluctuation in GDM patients was less than that in PGDM, so that the results may be better after individualized strategy. Hence, they had a higher acceptance of the disease than patients with GDM and could accept hypoglycemic treatment psychologically, which can improve the anxiety and quality of life of patients to some extent. However, we did not find any improvement in pregnancy-related anxiety in GDM patients in the CGM group, and we hypothesized that this improvement might be supported by larger sample size. Besides, we also indicated improvement in pregnancy-related anxiety in PGDM patients in the SMBG group, as PAQ scores correlated with glycemic parameters. In summary, blood glucose levels are related to the mental health of pregnant women, and good control of blood glucose can improve mental status.

**Figure 4:**
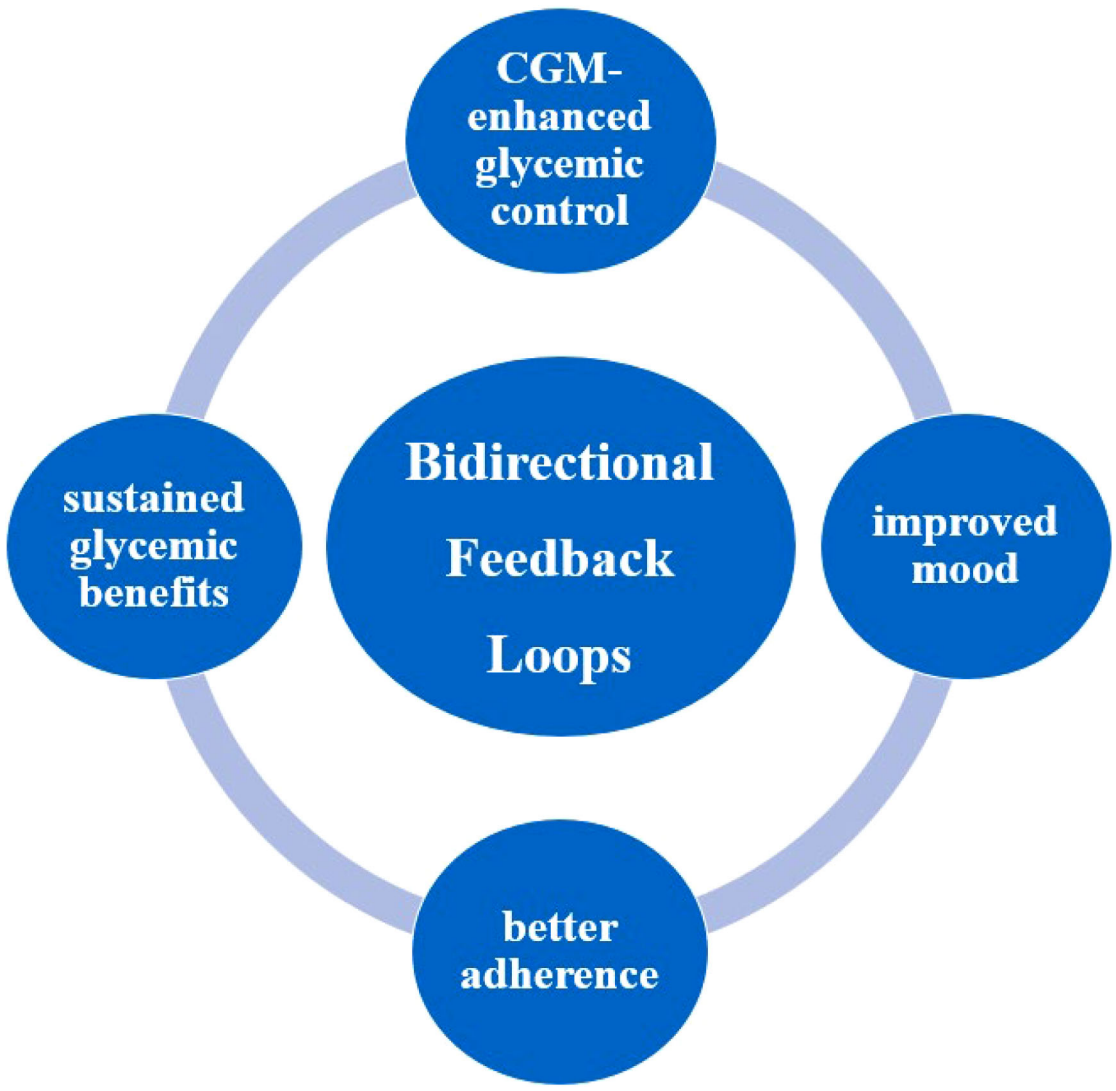
Bidirectional feedback loops.

The potential mechanisms underlying the effect of CGM on mental health could be expanded through hypothetical pathways. The proposed dual-pathway model integrates “physiological feedback”, “psychosocial mediators” and bidirectional feedback loops. 1) Physiological Feedback a. Glycemic Stability and Stress Response: The hypothalamic-pituitary-adrenal axis is thought to play a vital role in glucose homeostasis and diabetes. Stress reduces glucose and total cholesterol (TC) levels in female rats under the same behavioral tests (54). While human studies exhibit lower TIR with higher serum cortisol (P< 0.001) in T2DM patients (55). b. Neurotransmitter Modulation: CGM-driven hypoglycemia prevention preserves tryptophan availability for serotonin synthesis. Compared with the TIR-H (TIR > 70%) group, the TIR-L (TIR< 50%) group exhibits lower serum levels of 5-hydroxy-L-tryptophan and more (56). c. Psychosocial Mediators: CGM can enhance precise monitoring of diabetes symptoms associated with dysglycemia, diabetes-related complications, and mental conditions within the realm of precision medicine (57). 2) Pychosocial Mediators a. Self-Efficacy and Cognitive Liberation: CGM empowers patients to predict glycemic trends, reducing “decision fatigue” from frequent self-monitoring and enhancing confidence in “daily activities”. b. Anxiety Mitigation: Animals that have previously experienced recurrent hypoglycemia exhibit an increase in norepinephrine levels in the amygdala during hypoglycemia, accompanied by increased anxiety (58). 3) Bidirectional Feedback Loops: Emerging models suggest a virtuous cycle, CGM-enhanced glycemic control → improved mood → better adherence → sustained glycemic benefits (**Figure 4**).

The innovation of this study is that it is the first to explore the positive significance of individualized strategy combined with CGM on anxiety and diabetes specific quality of life in patients with DIP. The limitations of this study include: 1) This trial was a single-center study; 2) The sample size of this study was small, and the analysis of risk factors for anxiety and depression is limited; 3) The duration of the study was only 14 days to meet the clinical requirements of smoothly lowering blood glucose in the short term, and the improvement of blood glucose and partial psychological status was observed. However, if the individualized strategy combined with CGM was longer, its effect on the improvement of psychological status and quality of life might be more obvious, and its clinical significance on the physical and mental regulation of patients would be more significant.

Although this study has confirmed the short-term psychological benefits of CGM, several unresolved issues persist. Future research should focus on the following areas.

1) Extend the follow-up period to assess the persistence of psychological benefits. Future research should include long-term longitudinal studies (such as/e.g. 1–3 years postpartum) to determine whether the psychological protective effects of CGM are enduring and to explore whether they reduce the risk of postpartum depression; 2) Explore the Impact of CGM on Different Subgroups of DIG. It may impose a different psychological burden compared to GDM and PGDM (such as/e.g,T1DM or T2DM). Future studies should stratify the analysis of the differential impact of CGM on these subgroups and assess whether psychological support strategy need to be tailored accordingly; 3) Combine digital psychological intervention and optimize the clinical utility of CGM. Real-time data can be integrated with mobile health technology, such as developing an AI-based emotional warning system that provides immediate psychological counseling when abnormal blood glucose fluctuations are detected, or recommends relaxation training, thus forming a “blood glucose-psychological” dual management model; 4) Focus on the clinical significance of CGM beyond blood glucose control. Currently, the assessment of CGM primarily concentrates on metabolic indicators, including HbA1c, TIR, etc. Moving forward, a broader range of psychosocial indicators should be incorporated to comprehensively evaluate the clinical value of CGM.

In conclusion, the combination of individualized strategy and regular blood glucose monitoring (CGM or SMBG) enables DIP patients to achieve better blood glucose control in the short term and avoid the effects of hyperglycemia on the fetus and pregnant woman. As for the management of gestational diabetes, it is crucial to pay attention to the patient’s mental health along with the patient’s blood glucose level. CGM appears to be an effective tool for glycemic control and may contribute to improved mental health in DIP patients. A multidisciplinary approach, integrating endocrinology, obstetrics, and mental health support, is essential for optimizing DIP management. We call on researchers, clinicians, and policymakers to jointly advance the following actions. Incorporate mental health indicators into the clinical assessment system of CGM; Conduct multicenter, long-term follow-up studies to clarify the impact of CGM on postpartum mental states; Develop intelligent management tools that integrate CGM with psychological support to optimize the overall care model for DIP.

## 5 Conclusion

The individualized strategy combined with short-term glucose monitoring can positively impact glycemic improvement in the short term and meet the requirements of glycemic control in pregnancy, which has important clinical significance. The combined use of individualized strategy and CGM improves glycemic control and may have protective effects on psychological well-being.

## Data Availability

The original contributions presented in the study are included in the article/**Supplementary Material**. Further inquiries can be directed to the corresponding authors.
