# Supplementary material for: To assess the impact of individualized strategy and continuous glucose monitoring on glycemic control and mental health in pregnant women with diabetes
Source: Front Endocrinol (Lausanne). 2025 Jun 18;16:1470473. doi: 10.3389/fendo.2025.1470473 (PMC12213396; doi:10.3389/fendo.2025.1470473)
Supplement: Supplementary file 1 [file SupplementaryFile1.docx]

**How to interpret the scores**

The SAS and SDS scales have 20 items each, divided into 4 levels of scoring, including positive and negative integration questions. The total score is multiplied by 1.25 to obtain the standard total score.

A total score of less than 50 on the SAS scale is considered normal; A score of 50-60 indicates mild anxiety; Individuals with scores between 61 and 70 are classified as moderate anxiety A score of 70 indicates severe anxiety.

A total score of less than 53 on the SDS scale is considered normal; Individuals scoring 53-62 are classified as mild depression, those scoring 63-72 are classified as moderate depression, and those scoring above 72 are classified as severe depression.

The PAQ scale was developed by Chinese scholars and consists of 13 items, including concerns about fetal health, childbirth, and self-care. It is divided into 4 levels of scoring, ranging from "no worries" to "always worried", with scores of 1-4. Those with a total score of ≥ 24 have pregnancy related anxiety, while those with a total score of ＜ 24 have no pregnancy related anxiety. The higher the total score, the higher the level of pregnancy related anxiety in pregnant women.

The DSQL scale assesses the quality of life of pregnant women with diabetes from the four dimensions of physiology, psychology, social relations and treatment, a total of 27 items. Each item is scored 1-5 points in turn. The lower the total score, the better the quality of life.

## SAS

This scale consists of 20 questions. Please read the following content carefully and fill in according to the situation in the past week.

|  | No or very little time | small portion of time | considerable amount of time | the vast majority of time, or all time |
| --- | --- | --- | --- | --- |
| 1. I feel more anxious and nervous than usual | ⚪ | ⚪ | ⚪ | ⚪ |
| 2. I feel scared for no reason | ⚪ | ⚪ | ⚪ | ⚪ |
| 3. I am easily annoyed or feel scared | ⚪ | ⚪ | ⚪ | ⚪ |
| 4. I feel like I might go crazy | ⚪ | ⚪ | ⚪ | ⚪ |
| 5. I think everything is fine and nothing unfortunate will happen | ⚪ | ⚪ | ⚪ | ⚪ |
| 6. My hands and feet are trembling and trembling | ⚪ | ⚪ | ⚪ | ⚪ |
| 7. I am troubled by headaches, neck pain, and back pain | ⚪ | ⚪ | ⚪ | ⚪ |
| 8. I feel prone to weakness and fatigue | ⚪ | ⚪ | ⚪ | ⚪ |
| 9. I feel calm and composed, and it's easier for me to sit quietly | ⚪ | ⚪ | ⚪ | ⚪ |
| 10. I feel my heart beating very fast | ⚪ | ⚪ | ⚪ | ⚪ |
| 11. I suffer from waves of dizziness | ⚪ | ⚪ | ⚪ | ⚪ |
| 12. I have a fainting episode or feel like I'm about to faint | ⚪ | ⚪ | ⚪ | ⚪ |
| 13.I feel easy to exhale and inhale | ⚪ | ⚪ | ⚪ | ⚪ |
| 14. My hands and feet are numb and painful | ⚪ | ⚪ | ⚪ | ⚪ |
| 15. I am troubled by stomach pain and indigestion | ⚪ | ⚪ | ⚪ | ⚪ |
| 16. I often have to pee | ⚪ | ⚪ | ⚪ | ⚪ |
| 17. My hands are often dry and warm | ⚪ | ⚪ | ⚪ | ⚪ |
| 18. I blush and feel hot | ⚪ | ⚪ | ⚪ | ⚪ |
| 19. I easily fall asleep and sleep well | ⚪ | ⚪ | ⚪ | ⚪ |
| 20. I have nightmares | ⚪ | ⚪ | ⚪ | ⚪ |

## SDS

This scale consists of 20 questions. Please read the following content carefully and fill in according to the situation in the past week.

|  | No or very little time | small portion of time | considerable amount of time | the vast majority of time, or all time |
| --- | --- | --- | --- | --- |
| 1. I feel down and depressed | ⚪ | ⚪ | ⚪ | ⚪ |
| 2. I think the morning is the best time of the day | ⚪ | ⚪ | ⚪ | ⚪ |
| 3. I cry or feel like crying in waves | ⚪ | ⚪ | ⚪ | ⚪ |
| 4. I have poor sleep at night | ⚪ | ⚪ | ⚪ | ⚪ |
| 5. I eat as much as usual | ⚪ | ⚪ | ⚪ | ⚪ |
| 6. I am as happy as usual when I have close contact with the opposite sex | ⚪ | ⚪ | ⚪ | ⚪ |
| 7. I feel weight loss | ⚪ | ⚪ | ⚪ | ⚪ |
| 8. I have trouble with constipation | ⚪ | ⚪ | ⚪ | ⚪ |
| 9. My heart rate is faster than usual | ⚪ | ⚪ | ⚪ | ⚪ |
| 10. I feel tired for no reason | ⚪ | ⚪ | ⚪ | ⚪ |
| 11. My mind is clear as usual | ⚪ | ⚪ | ⚪ | ⚪ |
| 12. I don't think the things I often do are difficult | ⚪ | ⚪ | ⚪ | ⚪ |
| 13. I feel uneasy and unable to calm down | ⚪ | ⚪ | ⚪ | ⚪ |
| 14. I have hope for the future | ⚪ | ⚪ | ⚪ | ⚪ |
| 15. I am more easily excited than usual | ⚪ | ⚪ | ⚪ | ⚪ |
| 16. I think making a decision is easy | ⚪ | ⚪ | ⚪ | ⚪ |
| 17. I think I am a useful person and someone needs me | ⚪ | ⚪ | ⚪ | ⚪ |
| 18. My life is very interesting | ⚪ | ⚪ | ⚪ | ⚪ |
| 19. I think if I die, others will live better | ⚪ | ⚪ | ⚪ | ⚪ |
| 20. I am still interested in things that I am usually interested in | ⚪ | ⚪ | ⚪ | ⚪ |

## PAQ

Using the Likert 4-point scoring system, the scores range from 1 to 4 in order of severity, with a total score of 13 to 52. The higher the total score, the higher the level of pregnancy related anxiety.

|  | Not worried | occasionally worried | often worried | always worried |
| --- | --- | --- | --- | --- |
| 1. Are you worried that your child's gender may be contrary to the expectations of your family? | ⚪ | ⚪ | ⚪ | ⚪ |
| 2. Are you worried about not being mentally prepared for this pregnancy? | ⚪ | ⚪ | ⚪ | ⚪ |
| 3. Are you worried that this pregnancy will affect your job? | ⚪ | ⚪ | ⚪ | ⚪ |
| 4. Are you worried that pregnancy and childbirth may cause changes in your body shape? | ⚪ | ⚪ | ⚪ | ⚪ |
| 5. Are you worried that pregnancy will weaken your attractiveness to your husband? | ⚪ | ⚪ | ⚪ | ⚪ |
| 6. Are you worried that this pregnancy will bring financial pressure to your family? | ⚪ | ⚪ | ⚪ | ⚪ |
| 7. Are you concerned about the unhealthy condition of the fetus (such as deformities)? | ⚪ | ⚪ | ⚪ | ⚪ |
| 8. Are you worried about your child having intellectual disabilities? | ⚪ | ⚪ | ⚪ | ⚪ |
| 9. Are you concerned that your dietary habits may affect the fetus? | ⚪ | ⚪ | ⚪ | ⚪ |
| 10. Are you worried that your illness will affect the fetus? | ⚪ | ⚪ | ⚪ | ⚪ |
| 11. Are you worried about pain during childbirth? | ⚪ | ⚪ | ⚪ | ⚪ |
| 12. Are you worried about difficult delivery during childbirth? | ⚪ | ⚪ | ⚪ | ⚪ |
| 13. Are you concerned that your previous unhealthy lifestyle may have a negative impact on this pregnancy? | ⚪ | ⚪ | ⚪ | ⚪ |

## DSQL

Please read the following items and select the answer that best fits you in the past two weeks from the available options.

**Effect of diabetes on physiological function**

1. In general, how much does diabetes harm your health

⚪ No damage at all ⚪ Somewhat damaged ⚪ Moderate damage

⚪ Very damaging ⚪ Extreme damage

1. Do you often experience physical discomfort such as itching, numbness, and pain in your limbs

⚪ not at all ⚪ occasionally ⚪ About half the time

⚪ often ⚪ always

To what extent does the feeling of physical discomfort interfere with your life

⚪ There's no interference at all ⚪ It's a bit disruptive ⚪ moderate jamming

⚪ Very disruptive ⚪ Great interference

1. Do you feel that seeing things is becoming increasingly difficult

⚪ not at all ⚪ occasionally ⚪ About half the time

⚪ often ⚪ always

To what extent does the decline in vision affect your daily life

⚪ It has no impact at all ⚪ It has some impact ⚪ Moderate impact

⚪ Significant impact ⚪ Great impact

1. Do you feel that it is becoming increasingly difficult to hear others speak clearly

⚪ not at all ⚪ occasionally ⚪ About half the time

⚪ often ⚪ always

How much impact does hearing loss have on your daily life

⚪ It has no impact at all ⚪ It has some impact ⚪ Moderate impact

⚪ Significant impact ⚪ Great impact

1. Do you often feel chest pain, tightness, and palpitations

⚪ not at all ⚪ occasionally ⚪ About half the time

⚪ often ⚪ always

Do you feel that your skin and feet are prone to infection

⚪ not at all ⚪ occasionally ⚪ About half the time

⚪ often ⚪ always

To what extent do skin and foot infections affect your life

⚪ It has no impact at all ⚪ It has some impact ⚪ Moderate impact

⚪ Very influential ⚪ Great impact

1. Do you feel that your ability to respond to external things has decreased

⚪ It hasn't decreased at all ⚪ Somewhat decreased ⚪ Moderate decline

⚪ A significant decrease ⚪ Greatly decreased

1. Do you always feel hungry

⚪ not at all ⚪ occasionally ⚪ About half the time

⚪ often ⚪ always

**Psychological/Spiritual Dimensions**

1. Does diabetes often bring trouble and inconvenience to your daily life

⚪ not at all ⚪ occasionally ⚪ About half the time

⚪ often ⚪ always

Do you often think about what diabetes means to you

⚪ not at all ⚪ occasionally ⚪ About half the time

⚪ often ⚪ always

1. Are you worried that you might suddenly die

⚪ I'm not worried at all ⚪ Occasionally worrying ⚪ About half the time worrying

⚪ Frequently worried ⚪ Always worried

1. Does controlling your diet make you feel troubled

⚪ not at all ⚪ occasionally ⚪ About half the time

⚪ often ⚪ always

1. Do you think it's troublesome to regularly self test urine sugar or go to the hospital to check blood sugar

⚪ not at all ⚪ occasionally ⚪ About half the time

⚪ often ⚪ always

1. Do you feel nervous or uneasy due to diabetes

⚪ not at all ⚪ occasionally ⚪ About half the time

⚪ often ⚪ always

1. Are you satisfied with the current treatment effect

⚪ Extremely satisfied ⚪ Very satisfied ⚪ Moderate satisfaction

⚪ Not satisfied ⚪ Extremely dissatisfied

1. Do you believe that you can overcome the troubles of illness

⚪ I don't believe it at all ⚪ Somewhat believing ⚪ Moderate belief

⚪ I really believe it ⚪ I strongly believe

**Social relationship dimension**

21. In general, how much does diabetes damage your interpersonal relationship

⚪ No damage at all ⚪ Somewhat damaged ⚪ Moderate damage

⚪ Great damage ⚪ Extreme damage

22. Do you feel that you have been rejected because of diabetes

⚪ not at all ⚪ occasionally ⚪ About half the time

⚪ often ⚪ always

23. Does diabetes affect your status and role at home or at work

⚪ It has no impact at all ⚪ It has some impact ⚪ Moderate impact

⚪ Significant impact ⚪ Great impact

24. Do you often exchange experiences, problems and knowledge about diabetes with the patients around you

⚪ not at all ⚪ occasionally ⚪ About half the time

⚪ often ⚪ always

**Treatment dimension**

25. Have you experienced any adverse reactions such as nausea or allergies after taking medication

⚪ not at all ⚪ occasionally ⚪ About half the time

⚪ often ⚪ always

26. Have you experienced hypoglycemic reactions such as dizziness, sweating, and palpitations

⚪ not at all ⚪ occasionally ⚪ About half the time

⚪ often ⚪ always

To what extent does dietary control limit your lifestyle

⚪ There are no restrictions at all ⚪ There are some restrictions ⚪ Moderate restriction ⚪ Very restricted ⚪ Extremely restricted
